# New Organocatalytic Asymmetric Synthesis of Highly Substituted Chiral 2-Oxospiro-[indole-3,4′-(1′,4′-dihydropyridine)] Derivatives

**DOI:** 10.3390/molecules200915807

**Published:** 2015-08-31

**Authors:** Fernando Auria-Luna, Eugenia Marqués-López, Somayeh Mohammadi, Roghayeh Heiran, Raquel P. Herrera

**Affiliations:** Laboratorio de Organocatálisis Asimétrica, Departamento de Química Orgánica, Instituto de Síntesis Química y Catálisis Homogénea (ISQCH), CSIC-Universidad de Zaragoza, C/Pedro Cerbuna 12, E-50009 Zaragoza, Spain; E-Mails: 588861@unizar.es (F.A.-L.); mmaamarq@unizar.es (E.M.-L.); somayeh_babamohamadi@yahoo.com (S.M.); somaieheiran@gmail.com (R.H.)

**Keywords:** chiral base, enamine, isatylidene malononitrile, 1,4-dihydropyridine, enantioselective, isatin, organocatalysis, spirooxindole, 2-oxospiro-[indole-3,4′-(1′,4′-dihydropyridine)]

## Abstract

Herein, we report our preliminary results concerning the first promising asymmetric synthesis of highly functionalized 2-oxospiro-[indole-3,4′-(1′,4′-dihydropyridine)] via the reaction of an enamine with isatylidene malononitrile derivatives in the presence of a chiral base organocatalyst. The moderate, but promising, enantioselectivity observed (30%–58% ee (enantiomeric excess)) opens the door to a new area of research for the asymmetric construction of these appealing spirooxindole skeletons, whose enantioselective syntheses are still very limited.

## 1. Introduction

**Figure 1:**
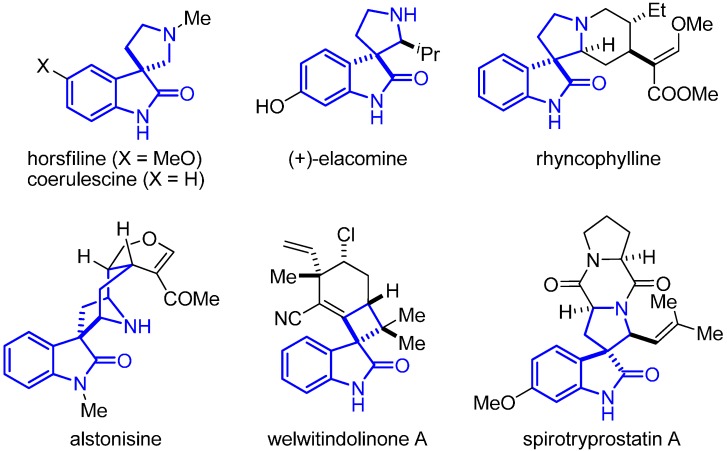
Representative structures of biologically-active spirooxindoles.

In the last few years, the development of new synthetic methods leading to spirooxindoles has aroused remarkable interest [1,2,3,4,5,6]. This structural motif can be found in natural and non-natural products, and its relevance is in part due to its challenging architecture, but also because many of these scaffolds exhibit interesting biological activity (Figure 1) [7,8,9,10,11,12]. Moreover, the potential of isatins to act both as an electrophile and as a nucleophile in many reactions and their easy availability have made them valuable building blocks in organic synthesis, attracting the attention of many scientists [13,14,15].

Moreover, 1,4-dihydropyridine derivatives are a significant class of heterocyclic compounds frequently found in natural products, and many of them also exhibit pharmacological properties [16,17,18,19,20]. As in other drugs, the role of the stereochemistry at C-4 can disclose both qualitative and quantitative differences in the biological activity. Thus, the control of the stereoselectivity in these chiral centers becomes an inspiring task of research, and therefore, there is growing interest for the development of enantioselective methods. Additionally, the generation of quaternary carbon centers is a very active and challenging area of investigation [21,22,23,24,25,26,27,28].

Thus, combining the interest and biological importance of spirooxindoles and 1,4-dihydropyridines and the search for new analogues with novel synergic properties, together with the increasing concern for sustainability, which makes essential the continuous search for new efficient catalytic procedures, encouraged us to explore a new route for the asymmetric synthesis of 2-oxospiro-[indole-3,4′-(1′,4′-dihydropyridine)] via asymmetric organocatalysis [29,30,31,32,33,34].

## 2. Results and Discussion

### 2.1. Hypothesis of Work

**Scheme 1:**
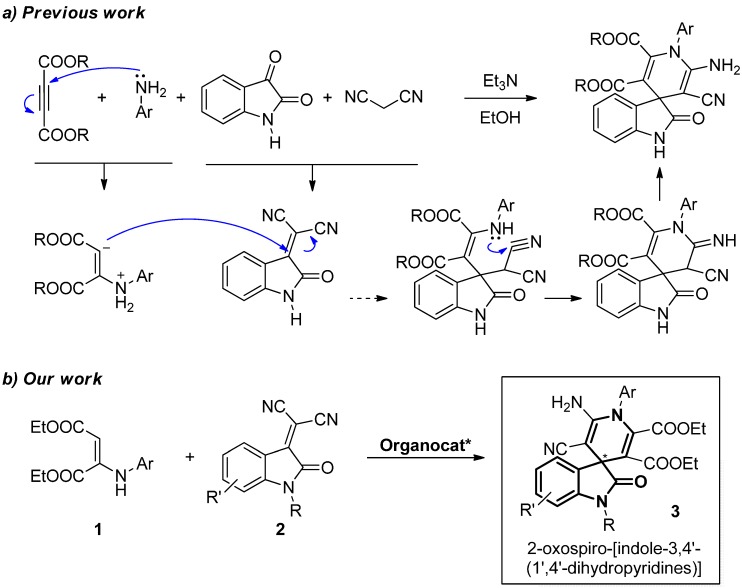
(**a**) Multicomponent synthesis of racemic 2-oxospiro-[indole-3,4′-(1′,4′-dihydropyridines)] and the mechanistic hypothesis in previous work; (**b**) enantioselective organocatalytic synthesis of enantioenriched 2-oxospiro-[indole-3,4′-(1′,4′-dihydropyridines)] **3** in our work.

As part of our ongoing research program about the synthesis of new chiral isatin derivatives, we focused our attention on 2-oxospiro-[indole-3,4′-(1′,4′-dihydropyridine)], in particular on its racemic synthesis reported by Perumal [35] and Yan [36]. Both groups used the same multicomponent strategy employing Et_3_N under reflux of EtOH (Scheme 1a). However, it is remarkable that Dabiri and Bazgir’s group obtained pyrano-fused spirooxindoles [37], instead of dihydropyridine-fused spirooxindoles, under very similar conditions [38,39].

The authors invoked a mechanism wherein a basic medium, isatin and malononitrile, condenses to give an intermediate that reacts with the enamine formed from the acetylenedicarboxylate and the amine (Scheme 1a). Based on this previous work and our experience with Brønsted bases as organocatalysts [40,41,42], we envisioned that a chiral organic base could promote this appealing and controversial reaction, starting directly from the preformed intermediates, enamines **1** and isatylidene malononitriles **2**, to give enantioenriched spirooxindoles **3** (Scheme 1b).

### 2.2. Synthesis of Starting Materials: Enamines ***1*** and Isatylidene Malononitriles ***2***

**Scheme 2:**
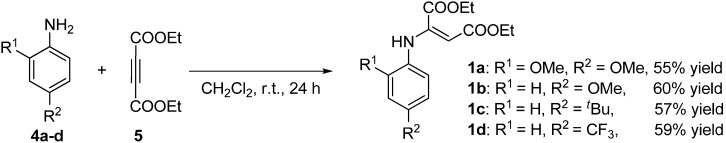
Preparation of the enamines **1a**–**d**.

For this purpose, we firstly synthesized four different enamines **1a**–**d** in one synthetic step, as described in Scheme 2 [43,44].

**Scheme 3:**
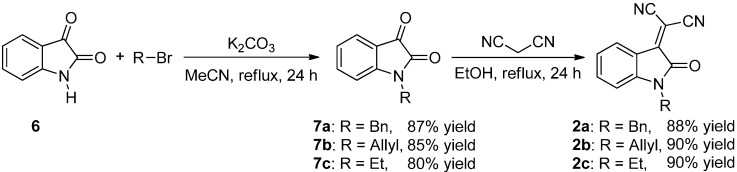
Synthesis of the *N*-protected 2-(2-oxoindolin-3-ylidene)malononitriles **2a**–**c**.

The synthesis of three differently-protected isatylidene malononitriles **2a**–**c** was also accomplished (Scheme 3), since the protection of the isatins has been found to be important in the reactivity and enantioselectivity of different processes [13,14,15].

The syntheses are performed in two steps: first, with the protection of the isatin **6** and, then, a Knoevenagel condensation with malononitrile, affording very good yields for each step, after column chromatography.

### 2.3. Screening

**Figure 2:**
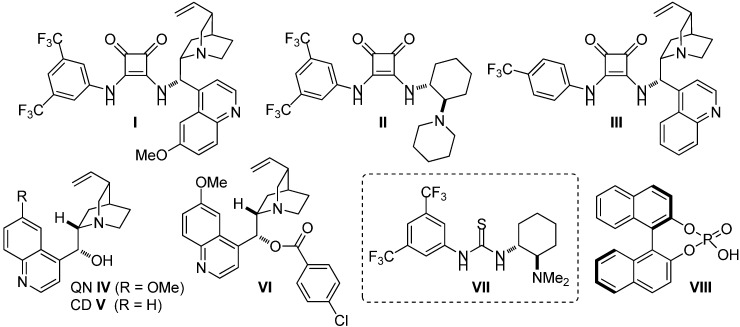
Model organocatalysts tested (**I**–**VIII**).

To test the viability of our hypothesis, we studied the efficiency of different organocatalysts **I**–**VIII** (Figure 2) in a model reaction between enamine **1a** and isatylidene malononitrile **2a** (Table 1).

**Table 1:** Screening of catalysts **I**–**VIII** for the synthesis of chiral spirooxindole **3aa**. 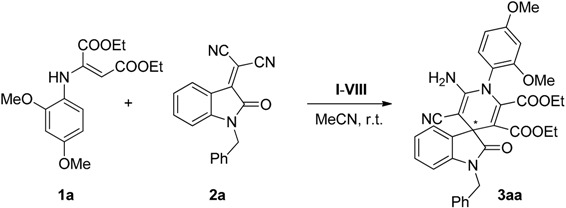

Entry	Cat. ^a^ (mol %)	1a (mmol)	2a (mmol)	MeCN (mL)	t (d)	yield (%) ^b^	ee ^c^ (%) ^d^
1	**I** (10)	0.2	0.1	1	5	15	Rac. ^e^
2	**II** (30)	0.12	0.06	0.3	5	3	4
3	**III** (30)	0.12	0.06	0.3	5	16	Rac. ^e^
4	**IV** (30)	0.2	0.1	1	5	77	5
5	**V** (30)	0.2	0.1	1	5	35	5
6	**VI** (30)	0.2	0.1	1	5	38	Rac. ^e^
7	**VII** (30)	0.2	0.1	1	5	30	42
8	**VIII** (30)	0.2	0.1	1	5	n.r. ^f^	n.d. ^g^

^a^ Catalyst; ^b^ isolated yields after column chromatography; ^c^ enantiomeric excess; ^d^ determined by chiral HPLC analysis (Daicel Chiralpak IB, Hex:EtOAc 6:4, 1 mL·min^−1^); ^e^ racemic mixture; ^f^ no reaction observed; ^g^ not determined.

As shown in Table 1, although the best reactivity was obtained with quinine (**IV**) (Entry 4), the most promising ee value was found with thiourea **VII**, known as Takemoto’s catalyst [45,46,47,48,49,50] (Entry 7). Interestingly, phosphoric acid **VIII** did not promote the reaction as expected, since the presence of a Brønsted base is believed to be crucial for the activation of this system, as previously reported [35,36] (Entry 8).

**Scheme 4:**
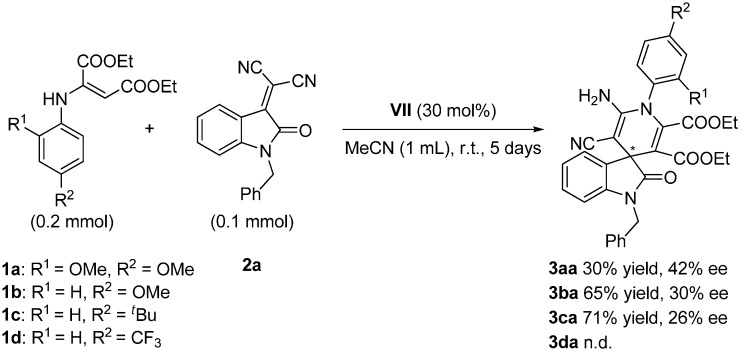
Effect of the substituents in the phenyl ring of the enamines **1a**–**d**.

The influence of the substituents of the aromatic ring in the enamine component **1**, over the reactivity and enantioselectivity of the process, was then considered in its reaction with benzyl-protected isatylidene malononitrile **2a** (Scheme 4).

The results suggest a clear influence of the electronic effects of the enamine ring **1** in both the reactivity and the enantioselectivity of the process, although the pattern of correlation is not clear at this point. Thus, while better reactivity was afforded with enamine **1c** (71% yield), the best enantioselectivity was reached with the dimethoxy substituted enamine **1a** (42% ee). In addition, untreatable reaction crude was observed with enamine **1d**.

**Scheme 5:**
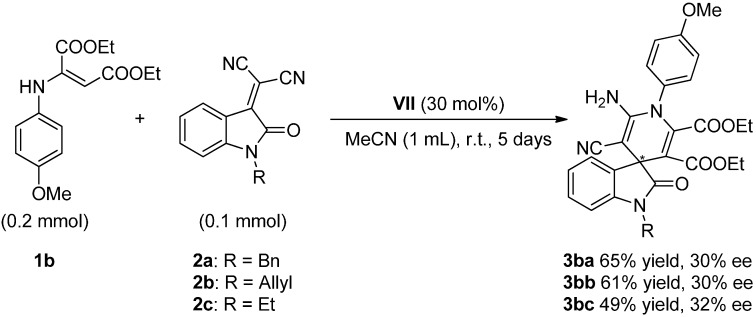
Influence of the protecting group on isatylidene malononitrile **2**.

Moreover, since the protecting group of the isatin scaffold can be relevant in the process, two additional protecting groups (allyl and ethyl) were tested in the reaction with enamine **1b** (Scheme 5).

Although the enantioselectivity of the process was similar in the three cases, the reactivity was slightly higher with the firstly used, *i.e.*, benzyl-protected isatylidene malononitrile **2a**.

**Figure 3:**
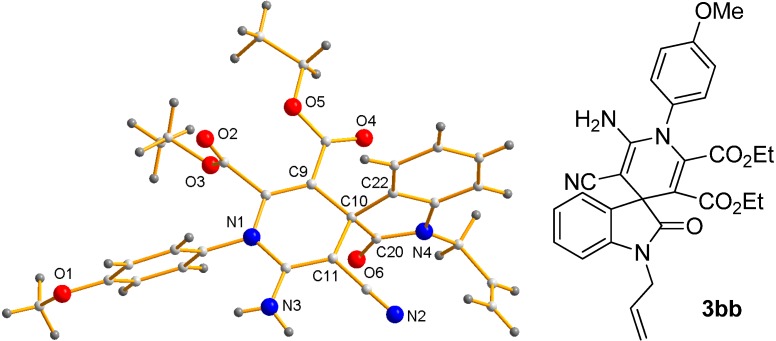
X-ray structure of adduct **3bb**.

Interestingly, an X-ray diffraction structure of compound **3bb** was obtained, and it is shown as evidence of the high complexity and functionalization of final target products (Figure 3) [51]. This structure is in agreement with the kind of molecules obtained by groups of Perumal and Yan through their multicomponent approaches [35,36].

**Figure 4:**
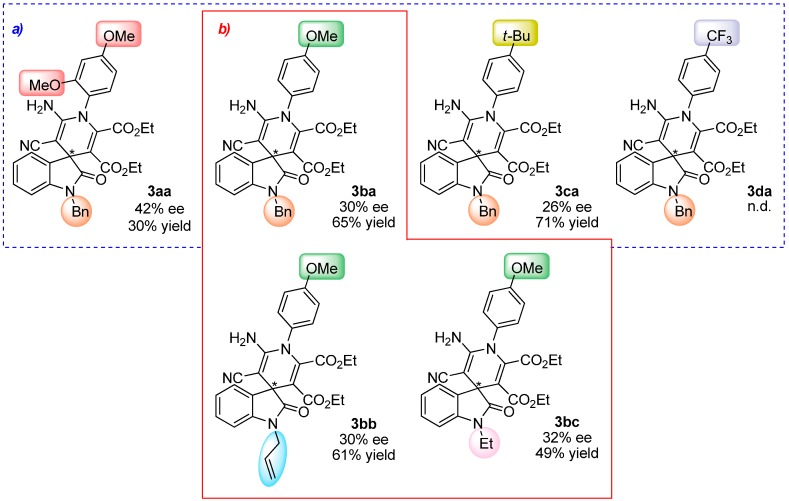
Summary of the comparative studies of (**a**) differently-substituted enamine **1** and (**b**) differently protected isatylidene malononitrile **2**.

Taking into account the above-mentioned results, summarized in Figure 4, we continued testing different key parameters, such as catalyst loading, concentration and solvent, with enamine **1a** and isatylidene malononitrile **2a**, which afford the best value of enantioselectivity in the final product (42% ee) using 30 mol % of catalyst **VII** (Table 2).

**Table 2:** Additional screening of the reaction. 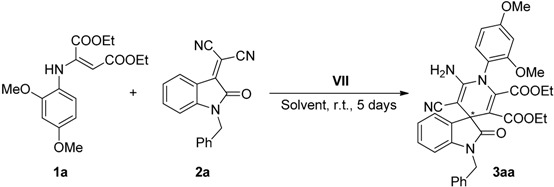

Entry	VII (mol %)	Solvent (mL)	yield (%) ^a^	ee (%) ^b^
1	30	MeCN (1)	30	42
2	20	MeCN (1)	25	23
3	10	MeCN (1)	29	11
4	30	MeCN (0.5)	55	46
5	30	EtOH (0.5)	12	14
6	30	EtOAc (0.5)	31	20
7	30	THF (0.5)	13	16
8	30	Toluene (0.5)	29	14
9	30	CH_2_Cl_2_ (0.5)	49	26
10	30	CHCl_3_ (0.5)	48	16

^a^ Isolated yields after column chromatography; ^b^ determined by chiral HPLC analysis (Daicel Chiralpak IB, Hex:EtOAc 6:4, 1 mL·min^−1^).

We firstly analyzed the effect of the catalyst loading (Entries 1–3), and no improvement was found lowering the amount of catalyst to 20 and 10 mol %. Then, we concentrated the reaction medium, getting slightly improved results (Entry 4). The exploration of the solvents (Entries 4–10), which was performed at the latter concentration, showed MeCN to be the best solvent in this case (Entry 4). As a conclusion, the best reaction conditions of those explored in this work were found to be 30 mol % of catalyst **VII** and 0.5 mL of MeCN as the solvent (Entry 4).

### 2.4. Scope of the Reaction

**Scheme 6:**
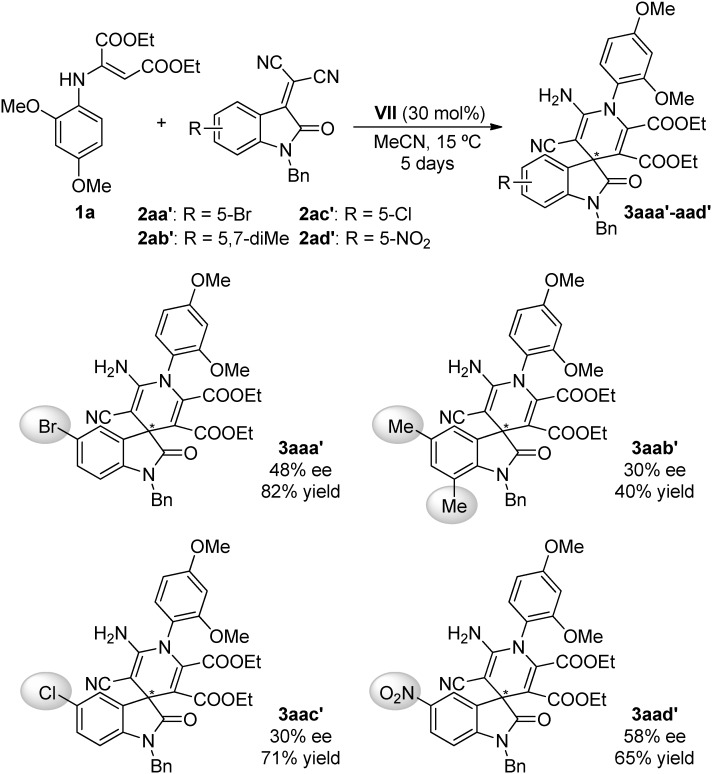
Scope of the organocatalyzed synthesis of 2-oxospiro-[indole-3,4′-(1′,4′-dihydropyridines)] **3aaa′**–**aad′**.

With the aim of exploring the generality of this reaction, various isatylidene malononitrile derivatives **2aa′**–**ad′** were studied under the optimized reaction conditions [52] (Scheme 6).

The final adducts **3aaa′**–**aad′** were obtained with moderate to good yield (40%–82%) and with moderate enantioselectivity (30%–58% ee). The results suggest the dependence of the reactivity of the process with the electronic properties of the aromatic ring of the isatin, since derivative **2ab′** (40% yield), with two methyl groups in its structure, was less reactive than those bearing an electron-withdrawing group in their structures (**2aa′**, **2ac′** and **2ad′** (65%–82% yield)) or the one without substituent (**2a** (55% yield)). In contrast, the enantioselectivity of the process seems to be independent of the electronic environment in the isatin skeleton.

### 2.5. Mechanism of the Reaction

**Scheme 7:**
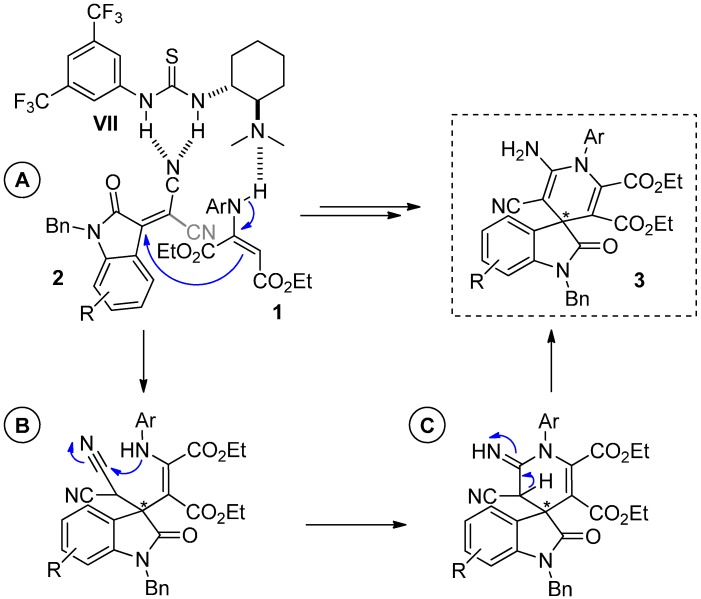
Plausible reaction mechanism. (**A**) Michael addition; (**B**) Intramolecular cyclization; (**C**) Tautomerization.

Based on the previous reported mechanism for the non-asymmetric version of this reaction [35,36] and our experimental results, we propose the tentative mechanism depicted in Scheme 7.

Initially, isatylidene malononitrile **2** would undergo a Michael addition with the enamine **1** in a concomitant coordination of both species with the catalyst **VII** (A). Then, an intramolecular nucleophilic addition of the NH to a nitrile group would close the piperidine ring in the intermediate (B). Final product **3** would be formed after a subsequent tautomerization of the intermediate (C) (Scheme 7). Although at this stage, we cannot ensure the bifunctional role for the catalyst in this system [53,54], the experimental results suggest that the presence of both moieties in the skeleton, thiourea and Brønsted base seems to be crucial for the success of this process. Additional studies are actually ongoing in our lab in order to shed light on the mechanism and with the aim of improving the enantiomeric excess values obtained so far.

## 3. Experimental Section

### 3.1. General Experimental Methods

Purification of reaction products was carried out by flash chromatography using silica gel (0.063–0.200 mm). Analytical thin layer chromatography was performed on 0.25 mm silica gel 60-F plates. ^1^H-NMR spectra were recorded at 300 and 400 MHz; ^13^C-APT-NMR spectra were recorded at 75 and 100 MHz; CDCl_3_ as the solvent. Chemical shifts were reported in the δ scale relative to residual CHCl_3_ (7.26 ppm) for ^1^H-NMR and to the central line of CDCl_3_ (77.0 ppm) for ^13^C-APT-NMR.

### 3.2. Materials

All commercially available solvents and reagents were used as received. Catalyst **I**, **II** and **III** were synthesized following our reported protocol [55], and the NMR spectra (^1^H-NMR and ^13^C-APT-NMR) for them are consistent with values previously reported in the literature: **I** [56], **II** [57] and **III** [56].

### 3.3. Synthesis and Physical, Analytical and Spectral data of Starting Materials *(**1*** and ***2**)* and the Final Compound *(**3**)*

#### 3.3.1. Synthesis of *E*-Enamines **1a**–**d**

To a mixture of diethyl 2-butynedioate **5** (5 mmol) in 40 mL of CH_2_Cl_2_, the appropriated aniline **4a**–**d** (10 mmol) was added at room temperature. The reaction vessel was covered with foil in order to prevent the decomposition of **5**. The reaction mixture was stirred 24 h at room temperature. Then, the solvent was evaporated under vacuum, and the reaction crude was purified by column chromatography (SiO_2_, Hex:EtOAc 85:15) (see Scheme 2).

*Diethyl 2-(2,4-Dimethoxyphenylamino)fumarate* (**1a**): Following the general procedure, compound **1a** was obtained as a yellow oil in a 55% yield. ^1^H-NMR (400 MHz, CDCl_3_) δ 1.06 (t, 3H, *J* = 7.1 Hz), 1.21 (t, 3H, *J* = 7.1 Hz), 3.69 (s, 3H), 3.72 (s, 3H), 4.07 (q, 2H, *J* = 7.1 Hz), 4.11 (q, 2H, *J* = 7.1 Hz), 5.22 (s, 1H), 6.30 (dd, 1H, *J* = 8.6 Hz, *J* = 2.6 Hz), 6.38 (d, 1H, *J* = 2.6 Hz), 6.70 (d, 1H, *J* = 8.7 Hz), 9.41 (s, 1H). ^13^C-APT-NMR (100 MHz, CDCl_3_) δ 13.8 (1C), 14.4 (1C), 55.5 (1C), 55.5 (1C), 59.7 (1C), 61.7 (1C), 91.0 (1C), 98.2 (1C), 103.8 (1C), 122.3 (1C), 122.9 (1C), 149.2 (1C), 152.5 (1C), 157.6 (1C), 164.2 (1C), 169.8 (1C).

*Diethyl 2-(4-Methoxyphenylamino)fumarate* (**1b**): Following the general procedure, Compound **1b** was obtained as a yellow oil in a 60% yield [58].

*Diethyl 2-(4-tert-Butylphenylamino)fumarate* (**1c**): Following the general procedure, compound **1c** was obtained as a yellow oil in a 57% yield. ^1^H-NMR (400 MHz, CDCl_3_) δ 0.97 (t, 3H, *J* = 7.1 Hz), 1.20 (t, 3H, *J* = 7.1 Hz), 1.20 (s, 9H), 4.06 (q, 2H, *J* = 7.1 Hz), 4.10 (q, 2H, *J* = 7.1 Hz), 5.24 (s, 1H), 6.77 (dt, 2H, *J* = 8.4 Hz, *J* = 1.8 Hz), 7.20 (dt, 2H, *J* = 8.6 Hz, *J* = 2.0 Hz), 9.57 (s, 1H). ^13^C-APT-NMR (100 MHz, CDCl_3_) δ 13.6 (1C), 14.4 (1C), 31.4 (3C), 34.3 (1C), 59.8 (1C), 61.9 (1C), 92.8 (1C), 120.9 (2C), 125.9 (2C), 137.8 (1C), 147.3 (1C), 148.9 (1C), 164.4 (1C), 169.6 (1C).

*Diethyl 2-(4-(Trifluoromethyl)phenylamino)fumarate* (**1d**): Following the general procedure, compound **1d** was obtained as a yellow oil in a 59% yield. ^1^H-NMR (400 MHz, CDCl_3_) δ 1.16 (t, 3H, *J* = 7.1 Hz), 1.31 (t, 3H, *J* = 7.1 Hz), 4.21 (q, 4H, *J* = 7.1 Hz), 5.54 (s, 1H), 6.94 (d, 2H, *J* = 8.4 Hz), 7.52 (d, 2H, *J* = 8.4 Hz), 9.72 (s, 1H). ^13^C-APT-NMR (100 MHz, CDCl_3_) δ 13.7 (1C), 14.3 (1C), 60.3 (1C), 62.4 (1C), 97.0 (1C), 120.0 (2C), 126.3 (q, 2C, *J* = 3.79 Hz), 143.5 (1C), 146.8 (1C), 163.9 (1C), 169.2 (1C).

#### 3.3.2. Synthesis of Isatylidene Malononitriles **2a**–**c** and **2aa′**–**ad′**

##### Protection of Isatin (**6**)

**Scheme 8:**
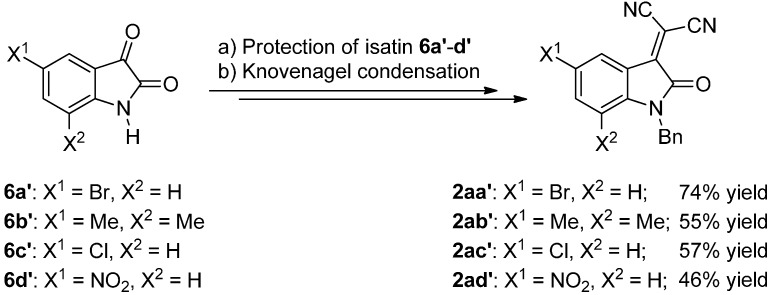
Synthesis of isatylidene malononitriles **2aa′**–**ad′**, starting from isatins **6a′**–**d′**.

To a mixture of the protecting reagent RBr (0.2 mmol) and K_2_CO_3_ (0.138 g) in MeCN (10 mL), isatin (**6**) was added (0.147 g) at room temperature. After that, the reaction mixture was stirred 24 h at reflux. Then, the solvent was evaporated under vacuum, and the reaction crude was purified by column chromatography (SiO_2_, Hex:EtOAc 8:2), giving rise to the corresponding product **7** (see Scheme 3 and Scheme 8).

##### Knoevenagel Condensation

To a mixture of **7** (1 mmol) in EtOH (10 mL), malononitrile (66 mg) was added. After that, the mixture was heated 24 h at reflux. Then, the solvent was evaporated under vacuum, and the reaction crude was purified by column chromatography (SiO_2_, Hex:EtOAc 8:2), giving rise to the corresponding product **2** (see Scheme 3 and Scheme 8).

The NMR spectra are consistent with the values previously published for **2a** [59], **2b** [60] and **2c** [61].

*2-(1-Allyl-2-oxoindolin-3-ylidene)malononitrile* (**2b**): Following the general procedure starting from isatin (**6**), compound **2b** was obtained as a black solid in a 77% overall yield. ^1^H-NMR (400 MHz, CDCl_3_) δ 4.38 (dt, 2H, *J* = 5.5 Hz, *J* = 1.6 Hz), 5.29–5.31 (m, 1H), 5.33–5.34 (m, 1H), 5.83 (ddt, 1H, *J* = 17.3 Hz, *J* = 10.1 Hz, *J* = 5.5 Hz), 6.86–6.90 (m, 1H), 7.16 (dt, 1H, *J* = 7.8 Hz, *J* = 0.9 Hz), 7.56 (dt, 1H, *J* = 7.8 Hz, *J* = 1.2 Hz), 8.15 (d, 1H, *J* = 7.9 Hz).

*2-(1-Benzyl-5-bromo-2-oxoindolin-3-ylidene)malononitrile* (**2aa′**): Following the general procedure starting from isatin **6a′**, compound **2aa′** was obtained as a black solid in a 74% yield. ^1^H-NMR (400 MHz, CDCl_3_) δ 4.91 (s, 2H), 6.68 (d, 2H, *J* = 8.5 Hz), 7.27–7.38 (m, 5H), 7.57 (dd, 1H, *J* = 8.5 Hz, *J* = 1.9 Hz), 8.21 (d, 1H, *J* = 1.8 Hz). ^13^C-APT-NMR (100 MHz, CDCl_3_) 44.3 (1C), 84.3 (1C), 110.2 (1C), 111.8 (1C), 112.1 (1C), 116.5 (1C), 119.6 (1C), 127.4 (2C), 128.5 (1C), 129.2 (3C), 133.6 (1C), 140.0 (1C), 144.9 (1C), 147.9 (1C), 162.0 (1C).

*2-(1-Benzyl-5,7-dimethyl-2-oxoindolin-3-ylidene)malononitrile* (**2ab′**): Following the general procedure starting from isatin **6b′**, compound **2ab′** was obtained as a black solid in a 55% yield. ^1^H-NMR (400 MHz, CDCl_3_) δ 2.22 (s, 3H), 2.29 (s, 3H), 5.16 (s, 2H), 7.05 (br s, 1H), 7.15 (d, 2H, *J* = 6.8 Hz), 7.26–7.38 (m, 3H), 7.86 (br s, 1H). ^13^C-APT-NMR (100 MHz, CDCl_3_) δ 18.5 (1C), 20.6 (1C), 45.3 (1C), 81.8 (1C), 111.0 (1C), 112.6 (1C), 119.2 (1C), 121.4 (1C), 125.2 (1C), 125.6 (2C), 127.8 (1C), 129.1 (2C), 133.8 (1C), 136.0 (1C), 142.3 (1C), 142.7 (1C), 148.8 (1C), 163.9 (1C).

*2-(1-Benzyl-5-chloro-2-oxoindolin-3-ylidene)malononitrile* (**2ac′**): Following the general procedure starting from isatin **6c′** [62,63], compound **2ac′** was obtained as a black solid in a 57% yield. ^1^H-NMR (300 MHz, CDCl_3_) δ 4.91 (s, 2H), 6.72 (d, 1H, *J* = 8.5 Hz), 7.27–7.39 (m, 5H), 7.42 (dd, 1H, *J* = 8.5 Hz, *J* = 2.1 Hz), 8.08 (d, 1H, *J* = 2.0 Hz). ^13^C-APT-NMR (75 MHz, CDCl_3_) δ 44.3 (1C), 84.3 (1C), 110.2 (1C), 111.7 (1C), 111.8 (1C), 119.2 (1C), 126.4 (1C), 127.4 (2C), 128.5 (1C), 129.2 (2C), 129.6 (1C), 133.7 (1C), 137.1 (1C), 144.5 (1C), 148.1 (1C), 162.2 (1C).

*2-(1-Benzyl-5-nitro-2-oxoindolin-3-ylidene)malononitrile* (**2ad′**): Following the general procedure starting from isatin **6d′** [64,65], compound **2ad′** was obtained as a red solid in a 46% yield. ^1^H-NMR (400 MHz, CDCl_3_) δ 5.00 (s, 2H), 6.94 (d, 1H, *J* = 8.8 Hz), 7.29–7.41 (m, 5H), 8.40 (dd, 1H, *J* = 8.8 Hz, *J* = 2.2 Hz), 8.99 (d, 1H, *J* = 2.1 Hz). ^13^C-APT-NMR (100 MHz, CDCl_3_) δ 44.8 (1C), 86.4 (1C), 109.8 (1C), 110.5 (1C), 111.3 (1C), 118.2 (1C), 122.0 (1C), 127.5 (2C), 128.9 (1C), 129.4 (2C), 132.5 (1C), 133.0 (1C), 144.0 (1C), 146.8 (1C), 150.1 (1C), 162.5 (1C).

#### 3.3.3. General Procedure for the Synthesis of Spirooxindoles **3**

To a mixture of catalyst **VII** (30 mol %, 12.4 mg) and enamine **1a** (0.2 mmol, 65 mg), in MeCN (0.5 mL), the isatin derivative **2** (0.1 mmol) was added. The reaction mixture was stirred 5 days at the indicated temperature. Then, the solvent was evaporated under vacuum, and the reaction crude was purified by column chromatography (SiO_2_, Hex:EtOAc 85:15), giving rise to the corresponding final adduct **3** (see Figure 4, Table 2 (Entry 4), and Scheme 6).

*Diethyl 2′-Amino-1-benzyl-3′-cyano-1′-(2,4-dimethoxyphenyl)-2-oxo-1′H-spiro[indoline-3,4′-pyridine]-5′,6′-dicarboxylate* (**3aa**): Following the general procedure (at room temperature), compound **3aa** was obtained as a brown solid in a 55% yield. The ee of the product was determined to be 46% by HPLC using a Daicel Chiralpak IB column (*n*-hexane:EtOAc 60:40, flow rate 1 mL·min^−1^, λ = 251 nm): τ_major_ = 27.9 min; τ_minor_ = 16.3 min. ^1^H-NMR (400 MHz, CDCl_3_) δ 0.73 (t, 3H, *J* = 7.1 Hz), 1.03 (t, 3H, *J* = 7.1 Hz), 3.62–3.74 (m, 1H), 3.84 (s, 3H), 3.84–3.93 (m, 3H), 3.96 (s, 3H), 4.28 (s, 2H), 4.79 (d, 1H, *J* = 15.7 Hz), 5.16 (d, 1H, *J* = 15.7 Hz), 6.50–6.55 (m, 2H), 6.67 (d, 1H, *J* = 7.7 Hz), 7.00–7.07 (m, 1H), 7.15 (dt, 1H, *J* = 7.7 Hz, *J* = 1.2 Hz), 7.22–7.36 (m, 4H), 7.39 (dd, 1H, *J* = 7.3 Hz, *J* = 1.1 Hz), 7.48–7.50 (m, 2H). ^13^C-APT-NMR (75 MHz, CDCl_3_) δ 13.4 (1C), 13.6 (1C), 44.6 (1C), 55.7 (1C), 56.3 (1C), 60.7 (1C), 61.8 (1C), 62.3 (1C), 99.7 (1C), 103.6 (1C), 104.7 (1C), 109.0 (1C), 116.4 (1C), 118.5 (1C), 123.0 (1C), 124.3 (1C), 127.5 (1C), 127.7 (2C), 128.6 (2C), 128.7 (1C), 132.2 (1C), 135.3 (1C), 135.7 (2C), 142.0 (1C), 144.6 (1C), 151.5 (1C), 158.3 (1C), 162.4 (1C), 162.7 (1C), 164.0 (1C), 178.0 (1C).

*Diethyl 2′-Amino-1-benzyl-3′-cyano-1′-(4-methoxyphenyl)-2-oxo-1′H-spiro[indoline-3,4′-pyridine]-5′,6′-dicarboxylate* (**3ba**): Following the general procedure (at room temperature), compound **3ba** was obtained as a brown solid in a 65% yield. The ee of the product was determined to be 30% by HPLC using a Daicel Chiralpak IC column (*n*-hexane:EtOAc 60:40, flow rate 1 mL·min^−1^, λ = 254 nm): τ_major_ = 6.7 min; τ_minor_ = 10.9 min. ^1^H-NMR (400 MHz, CDCl_3_) δ 0.64 (t, 3H, *J* = 7.1 Hz), 1.00 (t, 3H, *J* = 7.1 Hz), 3.57–3.65 (m, 1H), 3.85 (s, 3H), 3.87–3.92 (m, 3H), 4.28 (s, 2H), 4.83 (d, 1H, *J* = 15.7 Hz), 5.11 (d, 1H, *J* = 15.7 Hz), 6.70 (d, 1H, *J* = 7.7 Hz), 6.96–6.98 (m, 2H), 7.05 (dt, 1H, *J* = 7.6 Hz, *J* = 0.8 Hz), 7.17 (dt, 1H, *J* = 7.7 Hz, *J* = 1.3 Hz), 7.26 (tt, 1H, *J* = 7.3 Hz, *J* = 1.2 Hz), 7.31–7.40 (m, 5H), 7.47–7.49 (m, 2H). ^13^C-APT-NMR (75MHz, CDCl_3_) δ 13.3 (1C), 13.5 (1C), 44.5 (1C), 55.6 (1C), 60.7 (1C), 62.0 (1C), 62.2 (1C), 103.7 (1C), 109.0 (1C), 114.9 (2C), 118.2 (1C), 123.1 (1C), 124.1 (1C), 127.1 (1C), 127.5 (1C), 127.7 (2C), 128.7 (2C), 128.9 (1C), 131.7 (2C), 135.0 (1C), 135.7 (2C), 142.3 (1C), 144.1 (1C), 151.2 (1C), 160.9 (1C), 162.4 (1C), 164.0 (1C), 177.8 (1C).

*Diethyl 2′-Amino-1-benzyl-1′-(4-tert-butylphenyl)-3′-cyano-2-oxo-1′H-spiro[indoline-3,4′-pyridine]-5′,6′-dicarboxylate* (**3ca**): Following the general procedure (at room temperature), compound **3ca** was obtained as a brown solid in a 71% yield. The ee of the product was determined to be 26% by HPLC using a Daicel Chiralpak IA column (*n*-hexane:EtOAc 70:30, flow rate 1 mL·min^−1^, λ = 254 nm): τ_major_ = 10.6 min; τ_minor_ = 18.2 min. ^1^H-NMR (400 MHz, CDCl_3_) δ 0.64 (t, 3H, *J* = 7.1 Hz), 0.85 (t, 3H, *J* = 7.1 Hz), 1.34 (s, 9H), 3.61 (dq, 1H, *J* = 10.8 Hz, *J* = 7.1 Hz), 3.82–3.92 (m, 3H), 4.27 (s, 2H), 4.84 (d, 1H, *J* = 15.7 Hz), 5.11 (d, 1H, *J* = 15.7 Hz), 6.71 (d, 1H, *J* = 7.7 Hz), 7.06 (dt, 1H, *J* = 7.4 Hz, *J* = 0.8 Hz), 7.17 (dt, 1H, *J* = 7.7 Hz, *J* = 1.3 Hz), 7.26 (tt, 1H, *J* = 7.3 Hz, *J* = 1.2 Hz), 7.32–7.41 (m, 5H), 7.47–7.52 (m, 4H). ^13^C-APT-NMR (100 MHz, CDCl_3_) δ 13.3 (2C), 31.2 (3C), 35.0 (1C), 44.6 (1C), 60.7 (1C), 61.9 (1C), 62.3 (1C), 103.7 (1C), 109.0 (1C), 118.3 (1C), 123.1 (1C), 124.1 (1C), 126.8 (2C), 127.6 (1C), 127.7 (2C), 128.7 (2C), 128.9 (1C), 130.0 (2C), 132.1 (1C), 135.0 (1C), 135.7 (2C), 142.3 (1C), 143.9 (1C), 151.0 (1C), 154.2 (1C), 162.4 (1C), 164.0 (1C), 177.8 (1C).

*Diethyl 1-Allyl-2′-amino-3′-cyano-1′-(4-methoxyphenyl)-2-oxo-1′H-spiro[indoline-3,4′-pyridine]-5′,6′-dicarboxylate* (**3bb**): Following the general procedure (at room temperature), compound **3bb** was obtained as a brown solid in a 61% yield. The ee of the product was determined to be 30% by HPLC using a Daicel Chiralpak IC column (*n*-hexane:EtOAc 60:40, flow rate 1 mL·min^−1^, λ = 254 nm): τ_major_ = 7.8 min; τ_minor_ = 13.7 min. ^1^H-NMR (400 MHz, CDCl_3_) δ 0.79 (t, 3H, *J* = 7.1 Hz), 0.99 (t, 3H, *J* = 7.1 Hz), 3.73–3.81 (m, 1H), 3.85 (s, 3H), 3.86–3.93 (m, 3H), 4.21 (s, 2H), 4.22–4.27 (m, 1H), 4.53–4.61 (m, 1H), 5.25–5.29 (m, 1H), 5.42–5.48 (m, 1H), 5.86–5.96 (m, 1H), 6.86 (d, 1H, *J* = 6.8 Hz), 6.95–6.99 (m, 2H), 7.09 (dt, 1H, *J* = 7.5 Hz, *J* = 0.9 Hz), 7.26 (dt, 1H, *J* = 7.7 Hz, *J* = 1.3 Hz), 7.34–7.41 (m, 3H). ^13^C-APT-NMR (75 MHz, CDCl_3_) δ 13.4 (1C), 13.5 (1C), 42.9 (1C), 49.4 (1C), 55.7 (1C), 60.8 (1C), 62.0 (1C), 103.5 (1C), 108.9 (2C), 114.9 (1C), 117.9 (1C), 118.1 (1C), 123.1 (1C), 124.0 (1C), 127.0 (1C), 128.9 (1C), 131.3 (1C), 131.7 (2C), 134.9 (1C), 142.2 (1C), 144.0 (1C), 151.1 (1C), 160.9 (1C), 162.4 (1C), 163.9 (1C), 177.4 (1C).

*Diethyl 2′-Amino-3′-cyano-1-ethyl-1′-(4-methoxyphenyl)-2-oxo-1′H-spiro[indoline-3,4′-pyridine]-5′,6′-dicarboxylate* (**3bc**): Following the general procedure (at room temperature), compound **3bc** was obtained as a brown solid in a 49% yield. The ee of the product was determined to be 32% by HPLC using a Daicel Chiralpak IC column (*n*-hexane:EtOAc 60:40, flow rate 1 mL·min^−1^, λ = 254 nm): τ_major_ = 9.9 min; τ_minor_ = 16.5 min. ^1^H-NMR (400 MHz, CDCl_3_) δ 0.76 (t, 3H, *J* = 7.1 Hz), 0.98 (t, 3H, *J* = 7.1 Hz), 1.33 (t, 3H, *J* = 7.2 Hz), 3.68–3.78 (m, 2H), 3.83 (s, 3H), 3.84–3.95 (m, 4H), 4.24 (s, 2H), 6.84 (d, 1H, *J* = 7.8 Hz), 6.94–6.97 (m, 2H), 7.06 (t, 1H, *J* = 7.5 Hz), 7.25–7.29 (m, 1H), 7.32–7.38 (m, 3H). ^13^C-RMN (100 MHz, CDCl_3_) δ 12.5 (1C), 13.4 (1C), 13.5 (1C), 35.1 (1C), 55.6 (1C), 60.7 (1C), 62.0 (1C), 64.7 (1C), 108.1 (1C), 114.9 (2C), 118.0 (1C), 123.0 (1C), 124.1 (1C), 127.1 (1C), 129.0 (1C), 131.7 (2C), 135.3 (1C), 142.1 (1C), 144.1 (1C), 151.0 (1C), 160.9 (1C), 162.5 (1C), 164.0 (1C), 177.2 (1C).

*Diethyl 2′-Amino-1-benzyl-5-bromo-3′-cyano-1′-(2,4-dimethoxyphenyl)-2-oxo-1′H-spiro[indoline-3,4′-pyridine]-5′,6′-dicarboxylate* (**3aaa*′***): Following the general procedure (at 15 °C), compound **3aaa*′*** was obtained as a brown solid in an 82% yield. The ee of the product was determined to be 48% by HPLC using a Daicel Chiralpak IB column (*n*-hexane:EtOAc 60:40, flow rate 1 mL·min^−1^, λ = 256.4 nm): τ_major_ = 41.7 min; τ_minor_ = 15.9 min. ^1^H-NMR (300 MHz, CDCl_3_) δ 0.66 (t, 3H, *J* = 7.1 Hz), 0.96 (t, 3H, *J* = 7.2 Hz), 3.49–3.66 (m, 1H), 3.77 (s, 3H), 3.77–3.85 (m, 3H), 3.89 (s, 3H), 4.21 (br s, 2H), 4.71 (d, 1H, *J* = 15.7 Hz), 5.10 (d, 1H, *J* = 15.7 Hz), 6.44 (t, 1H, *J* = 9.8 Hz), 6.45 (t, 1H, *J* = 9.8 Hz), 6.60 (d, 1H, *J* = 7.8 Hz), 6.94–7.00 (m, 1H), 7.08 (dt, 1H, *J* = 7.7 Hz, *J* = 1.3 Hz), 7.18–7.34 (m, 4H), 7.42 (br d, 2H, *J* = 7.4 Hz). ^13^C-RMN (75 MHz, CDCl_3_) δ 13.4 (1C), 13.6 (1C), 44.6 (1C), 55.7 (1C), 55.7 (1C), 60.7 (1C), 61.8 (1C), 62.3 (1C), 99.6 (1C), 103.5 (1C), 104.7 (1C), 109.0 (1C), 116.3 (1C), 118.5 (1C), 123.0 (1C), 124.3 (1C), 127.7 (2C), 128.7 (2C), 128.7 (1C), 132.2 (1C), 135.3 (1C), 135.7 (1C), 141.9 (1C), 144.7 (1C), 151.5 (1C), 158.3 (1C), 162.4 (1C), 162.7 (1C), 164.0 (1C), 178.0 (1C).

*Diethyl 2′-Amino-1-benzyl-3′-cyano-1′-(2,4-dimethoxyphenyl)-5,7-dimethyl-2-oxo-1′H-spiro[indoline-3,4′-pyridine]-5′,6′-dicarboxylate* (**3aab*′***): Following the general procedure (at 15 °C), compound **3aab*′*** was obtained as a brown solid in a 40% yield. The ee of the product was determined to be 30% by HPLC using a Daicel Chiralpak IB column (*n*-hexane:EtOAc 60:40, flow rate 1 mL·min^−1^, λ = 257 nm): τ_major_ = 43.9 min; τ_minor_ = 22.4 min. ^1^H-NMR (400 MHz, CDCl_3_) δ 0.91 (t, 3H, *J* = 7.1 Hz), 1.03 (t, 3H, *J* = 7.2 Hz), 2.20 (s, 3H), 2.29 (s, 3H), 3.84–4.01 (m, 4H), 3.84 (s, 3H), 3.97 (s, 3H), 4.26 (br s, 2H), 5.04 (d, 1H, *J* = 16.8 Hz), 5.35 (d, 1H, *J* = 16.9 Hz), 6.48–6.55 (m, 2H), 6.75 (s, 1H), 7.090 (s, 1H), 7.21–7.42 (m, 6H). ^13^C-RMN (100 MHz, CDCl_3_) δ 13.6 (1C), 13.6 (1C), 18.6 (1C), 21.0 (1C), 45.9 (1C), 55.7 (1C), 56.2 (1C), 60.8 (1C), 61.8 (1C), 66.8 (1C), 99.7 (1C), 104.7 (1C), 116.5 (1C), 118.9 (1C), 119.0 (1C), 123.4 (1C), 126.3 (1C), 127.0 (2C), 128.7 (2C), 132.2 (1C), 132.3 (1C), 133.3 (1C), 136.3 (1C), 138.0 (1C), 144.3 (1C), 151.4 (1C), 158.3 (1C), 162.6 (1C), 162.7 (1C), 164.2 (1C), 179.2 (1C).

*Diethyl 2′-Amino-1-benzyl-5-chloro-3′-cyano-1′-(2,4-dimethoxyphenyl)-2-oxo-1′H-spiro[indoline-3,4′-pyridine]-5′,6′-dicarboxylate* (**3aac′**): Following the general procedure (at 15 °C), compound **3aac*′*** was obtained as a red solid in a 71% yield. The ee of the product was determined to be 30% by HPLC using a Daicel Chiralpak IB column (*n*-hexane:iPrOH = 70:30, flow rate 1 mL·min^−1^, λ = 244.1 nm): τ_major_ = 42.5 min; τ_minor_ = 22.9 min. ^1^H-NMR (300 MHz, CDCl_3_) δ 0.87 (t, 3H, *J* = 7.1 Hz), 1.03 (t, 3H, *J* = 7.1 Hz), 3.77–3.96 (m, 4H), 3.84 (s, 3H), 3.99 (s, 3H), 4.33 (br s, 2H), 4.73 (d, 1H, *J* = 15.8 Hz), 5.18 (d, 1H, *J* = 15.8 Hz), 6.49–6.59 (m, 3H), 7.09–7.12 (m, 1H), 7.24–7.47 (m, 7H). ^13^C-RMN (75 MHz, CDCl_3_) δ 13.6 (1C), 13.6 (1C), 44.7 (1C), 55.7 (1C), 55.7 (1C), 60.8 (1C), 60.9 (1C), 61.9 (1C), 99.5 (1C), 102.7 (1C), 104.6 (1C), 110.0 (1C), 115.8 (1C), 118.3 (1C), 124.9 (1C), 127.6 (2C), 128.1 (1C), 128.6 (1C), 128.7 (2C), 132.1 (1C), 135.2 (1C), 137.0 (1C), 140.2 (1C), 144.9 (1C), 151.5 (1C), 158.4 (1C), 162.2 (1C), 162.8 (1C), 163.7 (1C), 177.6 (1C).

*Diethyl 2′-Amino-1-benzyl-3′-cyano-1′-(2,4-dimethoxyphenyl)-5-nitro-2-oxo-1′H-spiro[indoline-3,4′-pyridine]-5′,6′-dicarboxylate* (**3aad*′***): Following the general procedure (at 15 °C), compound **3aad*′*** was obtained as a red solid in a 65% yield. The ee of the product was determined to be 58% by HPLC using a Daicel Chiralpak IB column (*n*-hexane:EtOAc 70:30, flow rate 1 mL·min^−1^, λ = 262.6 nm): τ_major_ = 62.9 min; τ_minor_ = 35.6 min. ^1^H-NMR (300 MHz, CDCl_3_) δ 0.85 (t, 3H, *J* = 7.1 Hz), 0.96 (t, 3H, *J* = 7.2 Hz), 3.75–3.94 (m, 4H), 3.78 (s, 3H), 4.03 (s, 3H), 4.30 (br s, 2H), 4.77 (d, 1H, *J* = 15.8 Hz), 5.16 (d, 1H, *J* = 15.8 Hz), 6.49–6.52 (m, 3H), 6.66 (d, 1H, *J* = 8.7 Hz), 7.17–7.42 (m, 6H), 8.06 (dd, 1H, *J* = 2.3 Hz, *J* = 8.6 Hz), 8.22 (d, 1H, *J* = 2.3 Hz). ^13^C-APT-RMN (75 MHz, CDCl_3_) δ 13.6 (1C), 13.6 (1C), 45.9 (1C), 55.7 (1C), 56.4 (1C), 61.2 (1C), 62.0 (1C), 63.1 (1C), 99.6 (1C), 102.1 (1C), 104.7 (1C), 108.7 (1C), 115.4 (1C), 118.0 (1C), 120.2 (1C), 126.0 (1C), 127.6 (2C), 127.6 (1C), 128.9 (2C), 132.0 (1C), 134.6 (1C), 136.3 (1C), 143.8 (1C), 145.3 (1C), 147.4 (1C), 151.6 (1C), 158.6 (1C), 161.9 (1C), 163.0 (1C), 163.6 (1C), 178.3 (1C).

## 4. Conclusions

In summary, we have developed an organocatalytic approach for the chiral formation of 2-oxospiro-[indole-3,4′-(1′,4′-dihydropyridine)] derivatives under mild conditions and operational simplicity. Final adducts were reached with promising results of enantioselectivity for the first time. Further mechanistic studies are required in order to understand and to prove the role of the used catalyst in this process. Moreover, additional studies with the aim of improving the enantioselectivity of the method are actively on-going in our laboratory.
